# The Role of Anesthetic Drugs in Liver Apoptosis

**DOI:** 10.5812/hepatmon.13162

**Published:** 2013-08-25

**Authors:** Ali Dabbagh, Samira Rajaei

**Affiliations:** 1 Anesthesiology Research Center, Shahid Beheshti University of Medical Sciences, Tehran, IR Iran; 2 School of Allied Medical Sciences, Tehran University of Medical Sciences, Tehran, IR Iran

**Keywords:** Liver, Apoptosis, Anesthesiology, Pharmacology

## Abstract

**Context:**

The modern practice of anesthesia is highly dependent ona group of anesthetic drugs which many of them are metabolized in the liver.

**Evidence Acquisition:**

The liver, of course, usually tolerates this burden. However, this is not always an unbroken rule. Anesthetic induced apoptosis has gained great concern during the last years; especially considering the neurologic system.

**Results:**

However, we have evidence that there is some concern regarding their effects on the liver cells. Fortunately not all the anesthetics are blamed and even some could be used safely, based on the available evidence.

**Conclusions:**

Besides, there are some novel agents, yet under research, which could affect the future of anesthetic agents' fate regarding their hepatic effects.

## 1. Context

Anesthesia is a modern human invention which was clinically introduced for the first time in October 1846 by William Morton, though the clinical effects of nitrous oxide had been discovered in 1844 (1).The introduction and utilization of anesthetic drugs has passed a long way, introducing newer generations of more effective drugs with less unwanted side effects; however, this process is not completed yet and the available anesthetic agents have their current side effects of course with a very low incidence (2).Liver is one of the main body organs performing drug metabolism among its many specific and unique functions. However, drug detoxification would create a spectrum of biochemical by-products imposed to the liver cells; while many pharmaceuticals, includingmost anesthetics, are metabolized, totally or partially, in the liver. This is why the cellular mechanisms for liver injury have a great impact in development and introduction of newer and more "liver friendly" anesthetic drugs.

**Figure 1.**
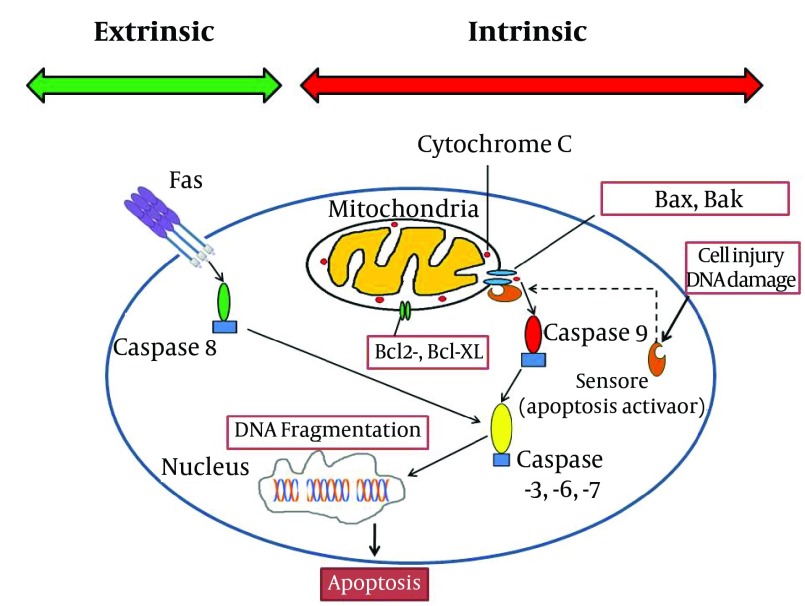
A General Schematic Presentation of the Apoptosis Pathway

Hepatic cells -during their metabolic functions- continuously produce reactive oxygen species. Reactive oxygen species are reduced to other forms of oxygen by mitochondria; this process may be deficient innonhealthyliver or when the liver is exposed to an extraordinary unwanted burden of toxins ( 3 - 6 ). This oxidant damage would disturb many parts of the cell structure in hepatocytes. Apoptosis (and not necrosis) is the main mechanism of liver injury, especially after drug related -including anesthetics- or viral injuries ( 6 - 14 ) at times ending in massive apoptosis ( 10 ). Apoptosis (programmed cell death) could be induced in two ways: intrinsic and extrinsic ( 9 , 11 ). Although both of pathways resulted in similar consequence (elimination of stressed cells), the initiation mechanisms are different. Intrinsic factors such as lack of growth mediators, DNA damage and cytoplasm detachment could accumulateproapoptoticmembers of Bcl-2 family (Bax and Bak) in mitochondrial membrane ( 4 , 9 , 10 , 15 , 16 ). This phenomenon could increase mitochondrial permeability and accomplish with displacement of cytochrome c and certain proapoptotic proteins from mitochondria to cytoplasm. These mediators activate caspases 9 ( 17 - 19 ) and other subsequent caspases ( 6 , 8 , 20 - 24 ). These enzymes induce DNA fragmentation, plasma membrane blebbing which finally result in formation of apoptotic bodies ( 7 , 20 ). Extrinsic pathway could be triggered by involvement of cell surface receptors which are consisted of a broad spectrum of death receptorsespeciallyFas (CD95) and TNF-RI ( 3 - 6 , 9 , 13 , 15 , 23 - 31 ). Activation of death receptors by related ligands induces recruitment of cytoplasmic adaptor proteins such as TRADD (TNF receptor-associated death domain) and FADD (Fas-associated death domain). This signal transduction would result in caspase 8 and subsequent cascade of caspases activation ( 4 , 15 , 17 , 18 , 23 , 24 , 28 , 29 ). Apoptotic bodies which formed after terminal activation of caspase 3 in both pathways are cleared by phagocytes without inducing inflammation ( 9 ). Liver cell structure remains the main location for the above interactions including the Kupffer cells, dendritic cells, natural killer (NK) cells, NKT cells, neutrophils, mast cells and T cells ( 4 , 12 - 14 , 16 , 27 , 32 - 38 ). The final fate of apoptosis cascade is determined by interaction between proapoptotic and antiapoptotic proteins in Bcl-2 family in cell structure (Figure 1) ( 4 , 9 , 15 , 23 , 32 , 39 , 40 ). 

## 2. Evidence Acquisition

### 2.1. The Effect of Anesthetic Drugs on Hepatic Apoptosis

During the recent years, it has been demonstrated in a great number of studies that most current anesthetic agents such as intravenous anesthetic agents (like ketamine, barbiturates, propofol, midazolam, diazepam, clonazepam), volatile agents (like halothane, isoflurane, desflurane, sevoflurane), xenon and even, muscle relaxants have been labeled as having apoptotic properties in animal studies, exerting their effect in a dose-dependent manner which its effects would be created as "time-dependent neurodegenerative effects especially in the developing animal brain"; while, a number of other studies have claimed these agents as being neuroprotective which preserve the brain tissue from unwanted adverse effects, like "apoptosis, degeneration, inflammation and energy failure"; however, one important point is that nearly all of these results are from animal models since performing such studies on the human brain is not an easy task (41-43). Sufficient proof (especially regarding lab evidence) for occurrence of neuroapoptosis in the developing brain is scarce yet, and the results gained from nearly all of the human studies demonstrate "associative, not causal relationships" (41, 42). This mandates further research clearing the possibility of previous animal findings in human.

On the other hand, there is a widespread research on the role of anesthetic agents in neuroapoptosis (41), mainly focusing on the neonatal brain; however, the possible role of anesthetic agents in liver apoptosis is not very clear yet and also, would it be real, it is not age limited as it is seen in neonatal apoptosis. Previous assessments have demonstrated that the deeper the level of anesthesia, the more severe the resultant neuroapoptosis; for example, a combined "nitrous oxide and isoflurane, or ketamine and propofol" would result in much severe neuroapoptosis compared toany single anesthetic administered alone (44).The anesthetic agents are many and diverse; however, according to a general classification used in many anesthesia texts and also, in this manuscript, the anesthetics could be classified as 4 groups according to their anesthetic function: hypnotics, analgesics, amnestic agents, and muscle relaxants.

## 3. Results

### 3.1. Hypnotics

The hypnotic agents with apoptotic activity could be classified as two main groups: N-methyl-D-aspartate (NMDA) antagonist, like ketamine; and gamma-aminobutyric acid (GABA) receptor agonists, like propofol or thiopental (45-50). Of course, hypnotic agents and amnestic agents have some overlapped clinical use; but we have discussed them under two different subtitles.

a)Ketamine: Among the intravenous hypnotics, there are many studies discussing the apoptotic effects of ketamine; especially potentiating hepatic apoptosis. Among all the proposed mechanisms for ketamine apoptosis, the most main ones are “up-regulation of NMDA receptors causing overestimation of glutamatergic system" and "hydroquinone toxicity" which is a metabolite of the drug (51). Ketamine also could "suppress phosphorylated extracellular signal-regulated protein kinase" (52)and "induce the formation of hyperphosphorylated tau", a "hallmark of Alzheimer's disease". S-(+)-ketamine, one of the main ketamine isomers creates "apoptosis in human HepG2 cells" causing hepatocyte and Kupffer cell injury; the potential ketamine injury occurs much sooner than pentobarbital injury (20, 53). Chronic ketamine use has been demonstrated to create hepatic cellular damage; while it did not affect the intestinal mucosa as much as the liver (54); also, ketamine would cause much more severe liver fibrosis. It could be concluded that adverse apoptotic effects of ketamine are seen more frequently when administered concomitantly with other anesthetics like benzodiazepines or when ketamine is used as a chronic drug; often as an abused drug; so, ketamine added to lidocaine could increase the apoptotic effects of lidocaine in an additive manner; some drugs like clonidine might have potential effects in prevention of apoptotic effects of ketamine (55-63).

b) Thiopental: is the prototype barbiturate used for anesthesia with an apoptotic mechanism by GABA-A agonistic action (64); also, through inducing lymphocyte death by "a CD95-independent mechanism" (65) and by "attenuated staurosporine-induced apoptosis and caspase-like activity" (66, 67); of course, the latter effect might be cardioprotective and against cardiomyocyte apoptosis.

c) Propofol: is one of the most common intravenous anesthetic agents and might attenuate " caspase-3 activation"; so, attenuating apoptotic effects of some anesthetics (68); also, propofol might have hepatic protective effects by reducing “the population of apoptotic cells and Caspase-3 and PARP cleavage in hepatic L02 cells in a dose-dependent manner” (69); though there are controversies (30, 52).

d) Etomidate: another intravenous anesthetic agent having in vitro apoptotic and cytotoxic effects in leukemia RAW264.7 cells (70); currently, no animal or clinical evidence for hepatic apoptosis is available.

e) Alpha 2 adrenergic receptor agonists: are used primarily as adjuvant to hypnotics; two main drugs belong to this classification, clonidine and dexmedetomidine, the 2nd one is synthetic, both having antiapoptotic effects, possibly against ketamine and isoflurane. Dexmedetomidine may prevent isoflurane-induced apoptosis in brain and some other organs. In clinic, dexmedetomidine could attenuate apoptotic effects of isoflurane in a dose dependent manner through a number of proposed mechanisms though; there is some controversy (62, 71-76).

f) Volatile anesthetics: these hydrophobic halogenated inhalational agents are often used for general anesthesia. Being among the most common inhalational anesthetics, have potential protective and also unwanted effects. Development of neuroapoptosis in animal brain (41), potential hepatotoxicity (1, 77, 78), attenuation of "antioxidant activity in plasma and erythrocytes", inhibition of "apoptosis in neutrophils", increasing DNA breaks" and "cell death" are among their effects, “cytotoxic effects on treated tumor cells” having a time dependent manner (79-81). Volatile anesthetics have "genotoxicity, cytotoxicity or teratogenicity" effects (82).The adverse effects of anesthetic drugs on liver cells could be altered by the process of liver cell apoptosis; which might be triggered by "concurrent viral infection" which in turn may "inhibit cytochrome (CYP) 450 activities and activate the hepatic innate immune system to proapoptotic factors" (8).

Isoflurane related apoptosis is GABA-A independent and could be prevented by dexmedetomidine; however, some controversies exist; especially when considering the administration dose or concomitant use of other anesthetics especially NMDA antagonists or GABA agonist anesthetics: Isoflurane has been shown to create apoptosis in human neuroglioma cell line with clinically relevant concentrations; emulsified form of isoflurane has protective effects against liver injury through the hepatic levels of malondialdehyde (MDA) and superoxide dismutase, suggesting "inhibition of cell death and improvement of antioxidation in mitochondria" as the potential protective mechanisms (53, 83-87).

Halothane can create apoptosis in vivo and in vitro in liver; Halothane has cytotoxic effects on treated tumor cells in a "time and cell line dependent" and even with low doses could impair irreversibly the cell genome; although it does not directly interact with DNA; also, clinical doses of halothane in animal model could decrease the viability of cells, impair DNA, and trigger "stress-induced apoptosis"; both halothane anesthesia and epidural anesthesia in animal models could induce apoptosis with a fall in lymphocyte counts (8, 19, 80, 82, 88-92).

g) Xenon belongs to noble gas family and is really an ideal inhalational anesthetic in clinical setting with efficient and satisfactory anesthetic properties not only for liver but also for other main organs of the body; many of its features are unique among all the anesthetic agents. No unwanted hepatic effects of xenon are reported. It is "a high-affinity glycine-site NMDA receptor antagonist" with both cardio protective and neuroprotective properties unique for xenon. The unique properties of xenon are possibly related to its interaction regarding the "dopaminergic pathways". It may even suppress the unwanted effects of ketamine and nitrous oxide and the apoptogenic activity of isoflurane. The only problems of xenon are potential pulmonary hypertension and also, the cost of the drug due to the especial technology of anesthesia machine needed for delivery of xenon to patients; a full discussion of the xenon, regarding its merits and possible problems could be found elsewhere (93).

### 3.2. Analgesics

Opioids might trigger the apoptosis process in a different number of cells; although a few studies have claimed some controversies regarding the role of opioids in apoptosis; also, δ-opioid receptors which are very much frequently found in the cellular membranes of liver cells, have an important role in "oncogenesis and progression of hepatic neoplasia, viral hepatitis and hepatic cirrhosis"; besides, it has been demonstrated in liver cells that "activation of δ-opioid receptor" would result in inhibition of mitochondrial apoptotic pathway through specialized interactions" involving protein kinase C: in this process, the dose and the time of administration of opioidshavekey roles: "higher levels of endogenous opioids" was shown to "increase hepatocyte apoptosis" which might be related to decreased level of antioxidant defense system in liver cells; the following few paragraphs are based on the studies related to opioids (21, 22, 94-112):

a)Morphine: chronic and repeated opioid administration, especially morphine, could induce apoptosis in hepatocytes through the main opioid receptors; these repeated high doses could contribute to oxidative stress in the cells of liver, especially considering that blockade of opioid receptors could be in confrontation of Fas-induced hepatitis, strengthen the mice liver, and also repeated morphine doses might possibly be in relation with deterioration of the host defense chains in mice and rat; these findings are much more important when considering the possible role of opioids in hepatitis pathophysiology;though some controversies exist.

b) Met-enkephalin: it has been proposed that opioid growth factor (i.e. [Met(5)]-enkephalin) and its receptor have an important role in "endogenous pathways controlling cell growth"; the concentration of the opioid growth factor is higher in metastasis-positive human liver tissue than the normal liver tissue; liver met-enkephalin has been demonstrated to have antitumor activity.

c) Methadone: kills leukemia cells through an apoptotic mechanism through mechanisms independent of caspases accompanied with cellular depletion of ATP stores and a critical state in cell bioenergetics; methadone has also been demonstrated to have a similar role in the treatment of small cell lung carcinoma through apoptosis.

d) Fentanyl: is a potent synthetic opioid agonist which could trigger lymphocyte apoptosis in a time-dependent manner.

e) Sufentanil: its protective effects with an antiapoptotic mechanism and modulating Bax and Bcl-2 expressionhavebeen demonstrated and similar results with up-regulation of p-FADD resulting in antiapoptotic effects for other opioids are demonstrated.

f) Remifentanil: is among the opioid compounds used for anesthesia with a number of specialpharmacologic properties. Due to its specific method of metabolism, mainly through cholinesterase metabolism in plasma, it has an ultrashorthalf-life, necessitating its mode of use to be only through intravenous infusion. There are some studies demonstrating the antiapoptotic effects of remifentanil in CNS and myocardium. In one animal study, the anti-inflammatory andantiapoptoticeffects of remifentanil were hepatic protective in rat models; this study demonstrated that "pretreatment with remifentanil can attenuate liver injury both in vivo and in vitro"; also, this study demonstrated that this protective effect is produced through NOS production which is not related to the activation of "opioid receptors" but is maintained by "exhausting reactive oxygen species"; so, it seems that remifentanil has some specific features, regarding its effect pathways and the mode of metabolism (i.e. somewhat different from other anesthetics) that might have promising features for improved liver effects of opioids as experienced in other tissues.

### 3.3. Amnestic Agents

Among the amnestic agents, benzodiazepines are the most well-known ones used much frequently in the perioperative period and also, for sedation of patients in ICU wards or during invasive procedures; benzodiazepines have the potential to induce apoptosis in a number of different cell lines, both in vitro and in vivo; the unwanted apoptotic effects of benzodiazepines (especially in the liver) are more expressed when they are coadministered with other anesthetics like ketamine; other benzodiazepines have also well studied apoptotic effects; the apoptotic effects of benzodiazepines could be suppressed by administration of vitamin C; which could restore the cellular reservoirs of glutathione. Midazolam is the prototype of these agents in anesthesia. Midazolam has apoptotic effects with a dose dependent manner, which is independent of GABA receptor and leads to necrosis with increased plasma concentrations; though it has varying degrees of intensity between different benzodiazepine compounds; midazolam or ketamine when added to lidocaine could increase the apoptotic effects of lidocaine in an additive or subadditive manner (9, 18, 63, 64, 113-121).

### 3.4. Muscle Relaxants

Muscle relaxants are a classification of anesthetic drugs which are used for prevention of movements during surgery (i.e. akinesia during operationor inside ICU for improvement of assisted ventilation). However, it does not seem that these agents would express liver apoptosis (122-125):

a) Pancuronium: an old nondepolarizing long acting muscle relaxant is demonstrated to have apoptotic effects on "peripheral blood lymphocytes" when used at clinical concentrations.

b) Cisatracurium: an intermediate acting muscle relaxant; acrylate esters are produced during its metabolism and could induce oxidative stress which is a well-known and very potent triggering factor for apoptosis in human cell lines.

c) Neostigmine: a muscle relaxant reversal agent, which has not been demonstrated to have apoptotic activity.

## 4. Conclusions

Future opportunities and potential therapeutics for prevention of anesthetic agents-induced hepatic apoptosis.There are some studies that might help us direct future studies for prevention of anesthetics-induced apoptosis, including hepatic apoptosis.

a)Normal hepatocytes counteract apoptosis by neutralizing free oxygen species using agents like vitamin C and vitamin E; ferritin, glutathione, and a number of enzymes; as mentioned before, administration of vitamin C could suppress the apoptotic effects of benzodiazepines through "restoration of cellular glutathione reservoirs" (4, 118, 126-129).

b) Beta estradiol, a sex hormone being the predominant estrogen during the active female reproduction years; however, it is also observed in male blood as a product of testosterone; this hormone protects against neuroapoptosis by anesthetics; mainly by up regulating serum levels of one of the members of protein kinase B family (Akt); which would protect Brain-derived neurotropic factor (BDNF); anesthetics can cause BDNF imbalance in "cerebral cortex and thalamus in time-dependent fashion"; 17 beta-estradiol pretreatment is also demonstrated to protect the liver from injuries with "heat-shock protein 70 overexpression " mechanism; however, beta-estradiol might improve apoptosis possibly in liver cells (19, 130-132).

c) One of the main agents that could inhibit the apoptotic effects of anesthetic agents is melatonin which could inhibit the "mitochondria-dependent apoptotic" (133).

d) Alpha2A-adrenoceptors including clonidine could negatively regulate the expression of caspase-3 in the neonatal cerebral cortex; exerting their protective roles against anesthetics; so clonidine could be used both as an anesthetic adjuvant and an antiapoptotic agent; its analog, dexmedetomidine, can implement its protective roles to prevent against the apoptotic effects of some anesthetics like isoflurane; their possible role in liver could be the topic of many future researches (62, 71, 73-76, 134).

e) Lithium has been shown to effectively counteract the apoptotic effects of ketamine and propofol; hence lithium has the possible role in prevention of anesthetic induced apoptosis, possibly in brain or liver (52).

f) Xenon has been demonstrated to haveboth cardioprotective and neuroprotective properties which are unique properties of the gas and mimicking the natural cellular protection pathways through K(ATP) channels; also, it can prevent “isoflurane, nitrous oxide and ketamine” apoptotic activity; possible future protective effects of xenon for patients at risk of hepatic apoptosis needing anesthesia and surgery could be promising (93).

g) Heme oxygenase 1 has been shown to "improve neurologic outcome" in rats by "protecting neurons against apoptosis". Possibly Heme oxygenase 1 could be tried to see if it could be useful for prevention of hepatic cell apoptosis after anesthetic administration (135).

h) Antithrombin decreases hepatic injury through releasing calcitonin gene-related peptide or CGRP; CGRP improves hepatic cellular tolerance to cell injury, including apoptosis; CGRP can in turn increase the production of insulin-like growth factor-I (IGF-I); on the other hand, capsaicin, increases the release of CGRP, which might increase IGF-I production, and thereby reduce liver apoptosis(9, 21, 134-138).

i) Gamma-hydroxybutyrate has been shown to protect liver against injury through different mechanisms (138).

j) Pentapeptide V5, with its full name as "pentapeptide Val-Pro-Met-Leu-Lys, V5" is an antiapoptoticpolypeptide (120, 137) which has been shown to be liver protective.

k) Also, a number of pan-caspase inhibitors, namely IDN-6556 (N-[(1,3-dimethylindole-2-carbonyl)valinyl]-3-amino-4-oxo-5-fluoropentanoic acid) and IDN-1965 are in preclinical phase and have been shown to be liver protective andantiapoptotic; which may have some role in prevention of anesthetics-induced apoptosis (including liver) in future years (17, 136, 139-142).

l) Depletion of Kupffer cells from the liver increases the risk of hepatic injury (as seen after acetaminophen model hepatic damage), reduction in IL 6, IL 10, TNFα production and number of Kupffer cells are seen in this condition accompanied with averted activity of Fas/Fas ligand; possibly, these phenomena are attenuated byIbuprofen; though the exact mechanism is controversial; also, Cannabinoid CB2 agonists have similar role in protecting Kupffer cells, being possible antiapoptotic agents in liver (9, 34, 39, 127, 143-150).

m) Also, "exogenous administration of S-adenosyl-l-methionine" prevents hepatotoxicity by the same mechanism (151).

n) After exposure of the liver cells to the viral agents or haptens, there would be an increase in the interleukin 1-β and δ levels which demonstrate Toll like receptor 4 (TLR 4) involvements. TLR 4 could enhance the presence of TNF-αin the Kupffer cells after hepatic cell insults (6, 37, 53).
